# Effectiveness of remote monitoring for patients with a high risk of cardiovascular disease: a 12-month matched cohort study in primary care

**DOI:** 10.1093/ehjdh/ztag014

**Published:** 2026-01-22

**Authors:** Nicoline E van Hattem, Margot M Rakers, Eric G Hiddink, Saskia le Cessie, Just A H Eekhof, Frank den Heijer, Niels H Chavannes, Hendrikus J A van Os, Douwe E Atsma, Tobias N Bonten

**Affiliations:** Department of Public Health and Primary Care, Leiden University Medical Center, Albinusdreef 2, Leiden 2333 ZA, the Netherlands; National Health Living Lab (NeLL), Leiden University Medical Center, Leiden, the Netherlands; Department of Public Health and Primary Care, Leiden University Medical Center, Albinusdreef 2, Leiden 2333 ZA, the Netherlands; National Health Living Lab (NeLL), Leiden University Medical Center, Leiden, the Netherlands; Department of Public Health and Primary Care, Leiden University Medical Center, Albinusdreef 2, Leiden 2333 ZA, the Netherlands; National Health Living Lab (NeLL), Leiden University Medical Center, Leiden, the Netherlands; Stichting Health Base, Houten, The Netherlands; Department of Clinical Epidemiology and Department of Biomedical Data Sciences, Leiden University Medical Center, Albinusdreef 2, Leiden, The Netherlands; Department of Public Health and Primary Care, Leiden University Medical Center, Albinusdreef 2, Leiden 2333 ZA, the Netherlands; Department of Public Health and Primary Care, Leiden University Medical Center, Albinusdreef 2, Leiden 2333 ZA, the Netherlands; National Health Living Lab (NeLL), Leiden University Medical Center, Leiden, the Netherlands; Department of Public Health and Primary Care, Leiden University Medical Center, Albinusdreef 2, Leiden 2333 ZA, the Netherlands; National Health Living Lab (NeLL), Leiden University Medical Center, Leiden, the Netherlands; Department of Public Health and Primary Care, Leiden University Medical Center, Albinusdreef 2, Leiden 2333 ZA, the Netherlands; National Health Living Lab (NeLL), Leiden University Medical Center, Leiden, the Netherlands; Health Campus the Hague, Leiden University Medical Center, The Hague, The Netherlands; National Health Living Lab (NeLL), Leiden University Medical Center, Leiden, the Netherlands; Delft University of Technology, Faculty of Industrial Design Engineering, Delft, the Netherlands; Department of Cardiology, Leiden University Medical Center, Leiden, The Netherlands; Department of Public Health and Primary Care, Leiden University Medical Center, Albinusdreef 2, Leiden 2333 ZA, the Netherlands; National Health Living Lab (NeLL), Leiden University Medical Center, Leiden, the Netherlands

**Keywords:** Remote monitoring, Blood pressure control, Chronic disease management, Preventive care, Digital health, Risk stratification

## Abstract

**Aims:**

This study aimed to evaluate the effect of remote monitoring using the Cardiovascular Risk Management (CVRM)-Box on blood pressure control, weight management, medication prescriptions, and consultation frequency in primary care patients at high risk of cardiovascular disease (CVD).

**Methods and results:**

In this matched cohort study, patients with a > 5% 10-year CVD mortality risk in primary care (2020–2024) were compared to propensity score-matched controls over 12 months. The CVRM-Box included smartphone-connected devices (blood pressure monitor, weighing scale, activity tracker) linked to general practitioner electronic health records.

Compared to controls, the intervention group showed modest reductions in office-measured systolic {−1.1 mmHg [95% confidence interval (CI), −3.7 to −1.5]; *P* = 0.39} and diastolic blood pressure [−0.04 mmHg (95% CI, −1.6 to 1.5); *P* = 0.96]. Sensitivity analyses yielded similar results. However, CVRM-Box assessments showed reductions in systolic [−5.5 mmHg (95% CI, −7.6 to −3.3); *P* < 0.001] and diastolic blood pressure [−3.8 mmHg (95% CI, −5.1 to −2.4); *P* < 0.001]. The intervention group also experienced greater reductions in weight [−0.9 kg (95% CI, −1.6 to −0.2); *P* = 0.01] and body mass index [−0.3 kg/m² (95% CI, −0.5 to −0.01); *P* = 0.007]. Additionally, antihypertensive medication use increased [0.12 (95% CI, 0.06 to 0.23); *P* = 0.04], while consultation frequency decreased (rate ratio 0.82; *P* = 0.002).

**Conclusion:**

While office measurements showed no additional blood pressure reduction, CVRM-Box measurements demonstrated significant decreases. The intervention also improved target blood pressure achievement, promoted weight reduction, increased antihypertensive use, and reduced consultation frequency.

## Graphical Abstract

**Graphical Abstract:**
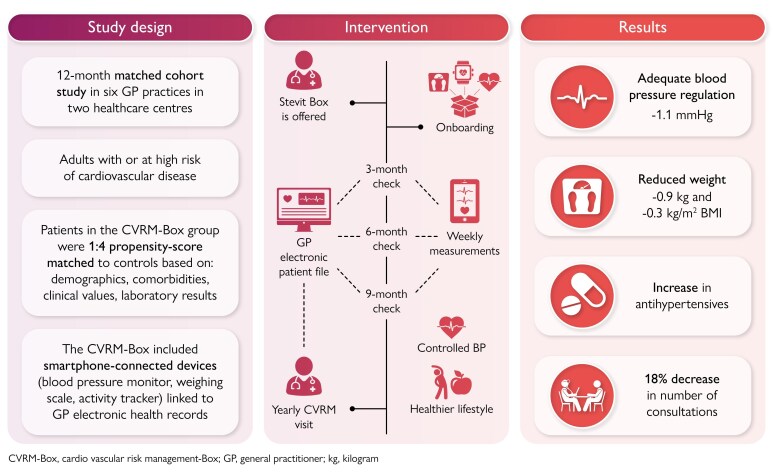

## Introduction

Although cardiovascular disease mortality has declined over recent decades, rising obesity rates, an ageing population, and healthcare staff shortages are increasing morbidity and healthcare burden.^1,2^ Therefore, exploring efficient and sustainable methods for delivering (preventive) care, such as remote monitoring of blood pressure, weight, and physical activity, is essential. These approaches help mitigate cardiovascular risk factors, including hypertension and overweight, while reducing strain on the healthcare system.^3–6^

Previous studies have shown that regular remote monitoring provides a more accurate reflection of blood pressure than sporadic office-based measurements.^7,8^ Additionally, randomized controlled trials (RCTs) have shown that remote monitoring has been shown to significantly reduce blood pressure in patients with poorly controlled (> 140/90 mmHg) hypertension compared to standard care.^5,6^ However, most eHealth interventions for high-risk primary care patients have focused primarily on hypertension management, overlooking other crucial aspects of cardiovascular risk, such as body weight. Moreover, their impact on healthcare provider workload is often not assessed.

The Cardiovascular Risk Management (CVRM)-Box represents a multi-component digital health infrastructure integrating various eHealth devices with a dedicated smartphone app for patients, securely connected to hospital and general practitioner (GP) electronic health record (EHR). In hospital care, The Box has successfully delivered high-quality, efficient care to patients after myocardial infarction, coronary bypass surgery, coronavirus disease-2019 (COVID-19), and other conditions.^9–11^ This study aims to examine the effect of remote monitoring using the CVRM-Box in a primary care setting to improve blood pressure control, weight management, medication prescriptions, and primary care consultation frequency.

## Methods

### Study design and setting

This matched cohort study was conducted from June 2020 to June 2024 among six primary care practices in healthcare centres in the Leiden region, The Netherlands. The healthcare centres are part of the data-driven innovation environment ‘Gezonde zorg, Gezonde regio’.^12^ The Medical Ethics Committee of the Leiden University Medical Center approved this study. All patients provided written online informed consent. All devices were CE medically certified. This study adheres to the Strengthening the Reporting of Observational Studies in Epidemiology (STROBE) reporting guideline for cohort studies^13^ (see Supplementary material online, *Table S1*).

### Patient selection

Patients enrolled in care programmes for CVRM or diabetes mellitus (DM), indicating a high risk of cardiovascular disease (defined as >5% 10-year mortality according to the SCORE Table),^14^ or those with established cardiovascular disease (acute coronary syndrome, percutaneous coronary intervention, stroke, and peripheral arterial disease) were eligible to participate in this study. Additional inclusion criteria included being 18 years or older, owning a smartphone, having internet access at home, and being proficient in Dutch. Exclusion criteria included pregnancy and unwillingness to sign informed consent. A maximum of 270 participants could be selected based on the availability of CVRM-Boxes within participating general practices and patients’ willingness to participate.

### Recruitment

Patients were recruited from six practices either during their annual CVRM consultation, where healthcare providers introduced the CVRM-Box, or through an information session at a local community centres. In both cases, patients could complete informed consent online and access detailed study information.

A control group was selected using propensity score matching in the Extramural LUMC Academic Network (ELAN) database, a regional integrative population-based data infrastructure.^15^ ELAN is a dynamic data infrastructure that integrates routine healthcare data from GPs, hospitals, and mental health services with municipal and national datasets from Statistics Netherlands. Established to support research on integrated patient care and health outcomes, ELAN covers 2.6 million residents of South Holland from 2007 onward, representing both urban and rural populations. The dataset is updated annually and retains key health information (e.g. mortality, medication, morbidity) even after individuals relocate. It includes coded data on clinical measures, laboratory results, diagnoses, prescriptions, healthcare utilization, and referrals.^15^ Eligible patients were enrolled in CVRM or diabetes care programmes, with correctly recorded blood pressure and body mass index (BMI) in their electronic files. A 1:4 propensity score matching was performed using logistic regression to calculate the propensity score and with nearest neighbour matching (R-studio*, MatchIt*) based on several baseline variables, including demographics (age, sex, social demographic status), comorbidities (DM, stroke, myocardial infarction), clinical values (systolic and diastolic blood pressure, BMI, number of antihypertensives), and laboratory results (eGFR, HDL, LDL, total cholesterol, HbA1c).

### Intervention

**Figure 1:**
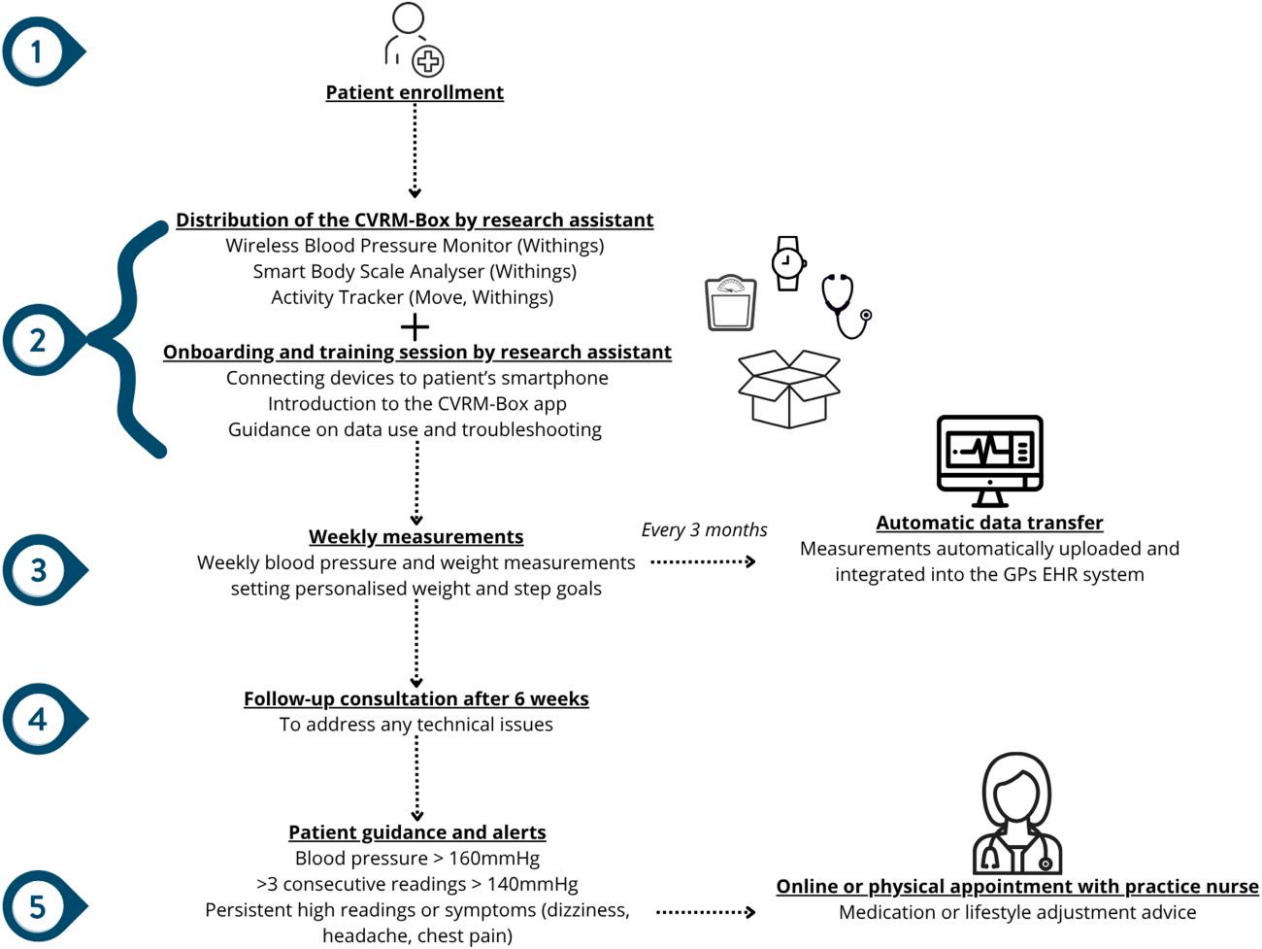
Flowchart intervention's content and structure.

Patients received the CVRM-Box containing connected devices, including a blood pressure monitor (Wireless Blood Pressure Monitor; Withings), a weighing scale (Smart Body Scale Analyzer; Withings), and an activity tracker (Move, Withings). Measurements were immediately shown in the patients’ Box app, directly transferred to, and integrated into the EHR (Medicom, PharmaPartners) of the GP practice. The practice nurse (PN) reviewed the patients’ average readings every three months (*Figure 1* and Supplementary material online, *Figure S1*).

To support patients in using the CVRM-Box at home, consultations with a research assistant were provided. These sessions focused on (1) connecting the wearables to the patients’ phones, (2) familiarizing them with the CVRM-Box app, and (3) answering any questions. Patients received instructions on performing weekly blood pressure and weight measurements and practical tips on reviewing their data. They could also set personalized weight and step goals. Patients were advised to contact the PN if their blood pressure readings exceeded 180 mmHg, if three consecutive readings were over 140 mmHg, if they noticed consistently high readings, or symptoms like dizziness, headaches, or chest pain. Follow-up consultations were held within six weeks after the distribution of the Box to address any technical issues.

### Outcomes

The primary outcomes were CVRM-Box and office-based blood pressure measurements over a 12-month period. Office-based blood pressure was recorded by a PN using a calibrated blood pressure monitor during the standard annual consultation. This included a baseline measurement, a follow-up after 12 months, and any additional measurements from interim consultations.

Secondary outcomes included the percentage of patients with controlled blood pressure (<140 mmHg) at the GP office after 12 months, changes in body weight measured by the CVRM-Box and at the GP practice, changes in physical activity measured by the CVRM-Box, changes in antihypertensive prescriptions [number and defined daily dose (DDD)], healthcare utilization measured by consultation frequency (defined as consultations within CVRM care) and the comparison of systolic blood pressure reduction between CVRM-Box and office-based measurements.

### Statistical analysis

Continuous data were presented as mean ± standard deviation (SD) or median with interquartile ranges (IQR) for baseline characteristics. Categorical variables are described as frequencies and percentages. Differences in proportions were tested for significance with an X^2^ test.

For CVRM-Box measurements, the baseline was defined as the average of the first two weeks of measurements, and 12-month values as the average of the last four weeks. A linear mixed-effects (LME) (R-studio version 4.2, *lme4*) model accounted for repeated measures, with random intercepts for GP practices and patients, and fixed effects for time and intervention. Covariates included sex, baseline blood pressure, baseline BMI, age, and cardiovascular disease history. An interaction term between time and intervention assessed treatment effects, and missing CVRM-Box data were imputed using last observation carried forward (LOCF), assuming stability of blood pressure over time and preventing overestimation of the Box’s effect. For the activity tracker data, only patients with at least four days of available measurements per week were included.^16^ According to the literature,^17^ data should be considered only when the device was worn for more than 10 h per day; therefore, a sub-analysis was conducted for HR Pulse users, as wear-time information was not available for the Withings Move. An LME was applied with time as a fixed factor, without additional covariates, and missing values were handled using the LOCF method.

For office-based measurements, the same LME model was used to compare the intervention and control groups. To assess the robustness of our findings, we conducted sensitivity analyses using different approaches to handle missing data, including complete case analysis, last observation carried forward (LOCF), and multiple imputation by chained equations (MICE). Additionally, interaction terms (time * intervention and subgroup) were used to evaluate the intervention's effect across subgroups: age (<70 vs. > 70 years), sex, baseline office systolic blood pressure (<140/90 vs. > 140/90 mmHg), and BMI (normal weight: BMI <25, overweight: BMI 25–30, and obesity: BMI >30).

For secondary outcomes, differences in weight and BMI between baseline and one year were assessed using a similar LME model, with adjustments for the same covariates, for both CVRM-Box and office measurements. Changes in the number of antihypertensive prescriptions and DDD from baseline to 12 months were analysed using linear regression. Consultation frequency was assessed with negative binomial regression and group differences were reported as adjusted rate ratios (RRs) with 95% CI. An exploratory analysis compared CVRM-Box and office-based measurements using a paired *t*-test in patients with both measurements at T0 and T12.

## Results

### Patient characteristics

**Figure 2:**
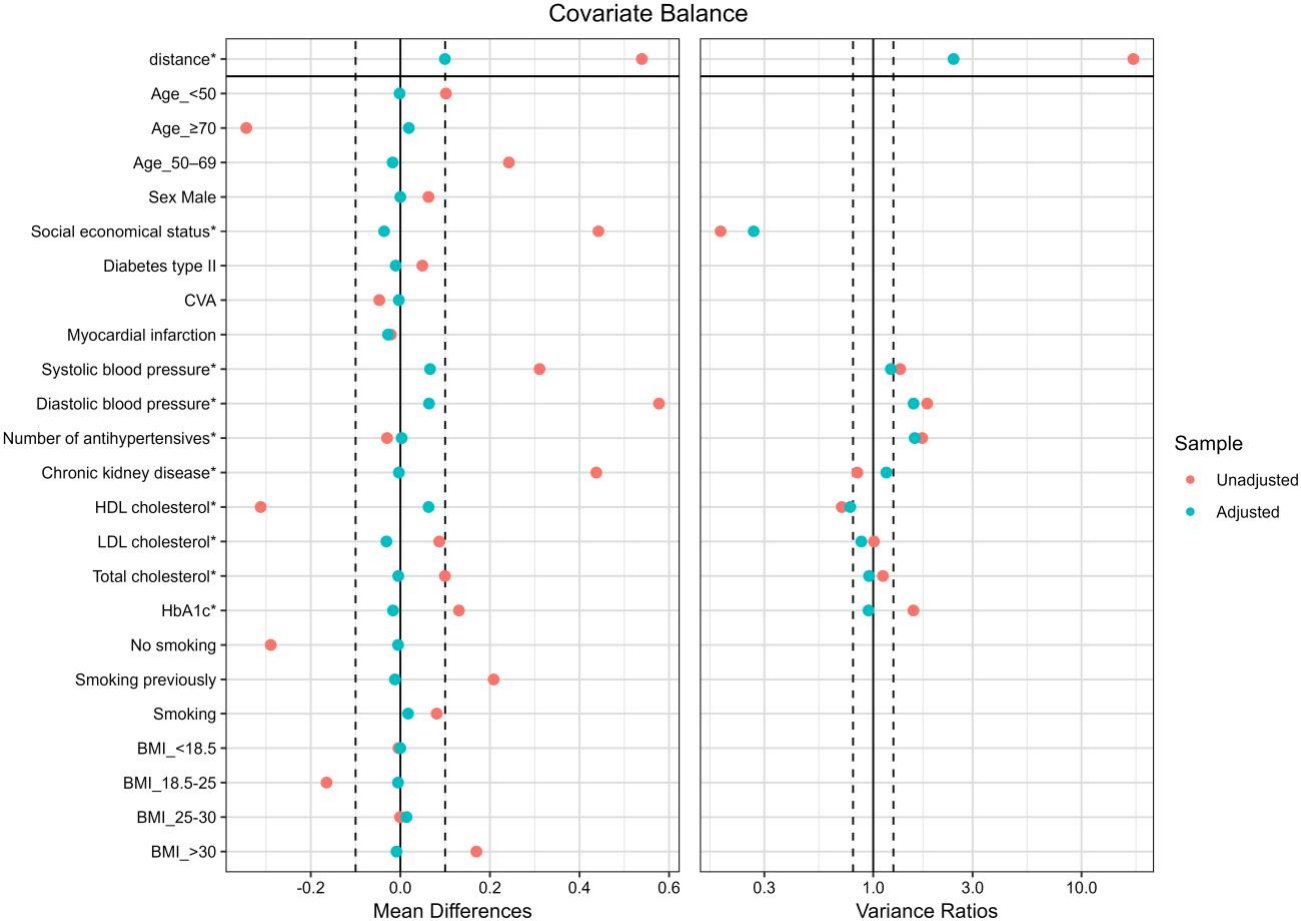
Balance plot propensity score matching.

Of the 2333 individuals assessed across six practices, 1832 (79%) were approached via email (89%) and during their yearly consultation (11%). A maximum of 270 patients could participate in the study. Among these, 26 (1%) withheld their consent (see Supplementary material online, *Figure S2*) resulting in 244 allocated to the intervention. The primary reasons for exclusion included connectivity or technical problems, 17 patients), the study being too burdensome (four patients), and no annual consultation at 12 months (seven patients). A total of 976 matched control patients were included for comparison. Baseline characteristics after the propensity score matching are summarized in *Table 1* and the corresponding balance plot is visualized in *Figure 2*. The mean (SD) age was 62.4 (11) years in the CVRM-Box group and 63.2 (10.4) years in the control group, with approximately 55% male participants. Mean baseline blood pressure was similar between groups (140/85 mmHg).

**Table 1:** Baseline characteristics in the propensity score-matched cohorts

Characteristics	Patients receiving the CVRM-Box (*n* = 244)	Matched controls (*n* = 976)	Standardised mean difference
**Age,** mean ± SD	62.4 ± 11.0	63.2 ± 10.4	0.04
**Sex,** *n* (%)			
Male	137 (56.4)	522 (53.4)	0.00
**Systolic blood pressure** ± SD	139.6 ± 16.4	139.5 ± 15.8	−0.01
**Diastolic blood pressure** ± SD	84.5 ± 11.0	84.5 ± 9.1	0.04
**BMI (kg/m^2^),** median (IQR)	30.2 (5.2)	29.7 (5.0)	
**Comorbidities**, *n* (%)			
Diabetes (type 2)	38 (15.6)	145 (14.8)	0.05
Stroke	12 (4.9)	47 (4.8)	0.06
Myocardial infarction	22 (9.1)	82 (8.4)	−0.04
Chronic kidney disease	21 (8.6)	88 (9.0)	0.01
**Smoking,** *n* (%)			
Current smoker	23 (9.5)	95 (9.7)	0.06
Used to smoke	87 (35.8)	329 (33.7)	−0.09
Never	130 (53.5)	553 (56.6)	0.05
**Number of antihypertensives** [median (IQR)]	1.00 [1.00, 2.00]	1.00 [1.00, 2.00]	−0.00

### Primary outcome; CVRM-box and office-based blood pressure measurements

#### Office-based measurements

After 12 months, the primary outcome data were available from 197 (81%) participants in the CVRM-Box group and 894 (92%) in the control group. In the intervention group, the mean systolic blood pressure measured at the primary office decreased from 139.8 ± 16.8 mmHg at baseline to 134.6 ± 15.0 mmHg at follow-up. In the control group, systolic blood pressure decreased from 139.5 ± 15.8 mmHg to 136.6 ± 14.8 mmHg, resulting in an adjusted mean difference of −1.1 mmHg [95% CI: 3.7 to 1.5; *P* = 0.39] (*Table 2*). There was no significant difference in the reduction of diastolic blood pressure between the CVRM-Box group and the control group after 12 months of follow-up [adjusted mean difference: −0.04 mmHg (95% CI: −1.6 to 1.5); *P* = 0.96]. Sensitivity analyses using complete case analysis, LOCF, and MICE procedures yielded similar results (see Supplementary material online, *Table S2*).

**Table 2:** CVRM-box and office-based mean blood pressure and weight at baseline and 12 months

	Baseline mean ± SD median [IQR}	12 months mean ± SD median [IQR}	mean difference (95% CI, *P*-value)	adjusted mean difference Box measurements (95% CI, *P*-value)	unadjusted mean difference office vs. usual care (95% CI, *P*-value)	adjusted^a^ mean difference office vs. usual care (95% CI, *P*-value)
**Systolic blood pressure (mmHg)**						
**CVRM-Box measurements**	138.9 ± 15.4; *n* = 231	131.8 ± 11.1; *n* = 144		− 5.5 (−7.6 to −3.3), *P* < 0.001^b^		
**Office-measurements**	139.8 ± 16.8; *n* = 218	134.6 ± 15.0; *n* = 197	− 4.7 (−7.1 to −2.2), *P* < 0.001; *n* = 180		− 2.1 (−4.7 to 0.4), *P* = 0.15	−1.1(−3.7 to 1.5), *P* = 0.39.
**Control group**	139.5 ± 15.8; *n* = 973	136.6 ± 14.8; *n* = 894	− 2.8 (−1.8 to −3.8), *P* < 0.001; *n* = 893			
**Diastolic blood pressure (mmHg)**						
**CVRM-Box measurements**	85.7 ± 9.7; *n* = 231	81.5 ± 7.1 *n* = 144		−3.8 (−5.1 to −2.4), *P* < 0.001^b^		
**Office-measurements**	84.5 ± 11.0; *n* = 218	81.7 ± 10.2; *n* = 197	− 2.4 (−4 to −0.8), *P* < 0.001, *n* = 180		− 0.7 (−2.2 to 0.8), *P* = 0.35	− 0.04 (−1.6 to 1.5), *P* = 0.96
**Control group**	84.5 ± 9.1; *n* = 973	82.5 ± 8.6; *n* = 894	−2.0 (−2.5 to-1.4), *P* < 0.001; *n* = 893			
**Weight (kg)**						
**CVRM-Box measurements**	90.1 ± 16.7; *n* = 229	86.2 ± 15.9; *n* = 132		−1.2 (−1.8 to −0.5), *P* < 0.001^b^		
**Office-measurements**	92.3 ± 17.2; *n* = 174	90.1 ± 16.1; *n* = 174	− 1.1 (−2.1 to −0.2), *P* = 0.02; *n* = 145		− 0.9 (−1.6 to 0.2), *P* = 0.01	− 0.9 (−1.6 to - 0.2), *P* = 0.01
**Control group**	89.1 ± 18.1; *n* = 947	88.9 ± 18.0; *n* = 898	− 0.3 (0.0 to −0.6), *P* < 0.001; *n* = 898			
**BMI (kg/m2)**						
**CVRM-box measurements**	29.5 ± 5.1 *n* = 207	28.0 ± 4.1; *n* = 119		− 0.4 (−0.6 to −0.1), *P* < 0.001^b^		
**Office-measurements**	30.2 ± 5.2; *n* = 244	29.7 ± 4.8; *n* = 160	− 0.4 (−0.7 to −0.1), *P* = 0.02; *n* = 144		− 0.3 (−0.6 to 0.1), *P* = 0.007	− 0.3 (−0.5 to −0.1), *P* = 0.007
**Control group**	29.7 ± 4.9; *n* = 947	29.5 ± 4.7; *n* = 855	− 0.1 (−0.2 to 0.0), *P* = 0.2; *n* = 828			
**Steps per day**						
**Steps accounting for days tracked**	4622 [3185–6269] *n* = 195	4063 [3128–6237] *n* = 112		298 (−597 to 20), *P* = 0.077^b^		
**Steps accounting for days tracked and hours worn**	4610 [3266– 519] *n* = 88	3819 [2870–5175		48 (− 442 to 590), *P* = 0.855^b^		

**IQR**, **interquartile range.**

^**a**^ **Adjusted for practices and measurements from the same participants, sex, baseline blood pressure, baseline BMI, age, and a history of CVD.**

^**b**^ **Imputated using last measurement carried forward**

However, the CVRM-Box group showed a larger improvement in the proportion of patients with controlled blood pressure compared to the control group over the 12-month period with proportions increasing from 52% to 64% in the intervention group and from 51% to 58% in the control group (*P* < 0.001).

**Figure 3:**
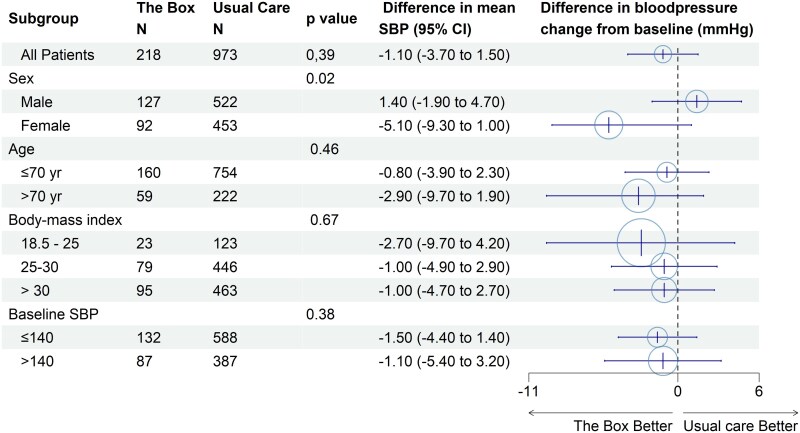
Forest plot of subgroup analyses for the change in systolic blood pressure from baseline to 12 months.

Subgroup analyses based on office-based measurements (*Figure 3*) indicated that women using the CVRM-Box experienced a greater effect size [−5.1 mmHg (95% CI: −9.3 to −1.0)] compared to men, who showed no benefit [1.4 mmHg (95% CI: −1.9 to 3.4); *P* = 0.02]. Patients with uncontrolled blood pressure who received the CVRM-Box demonstrated a greater reduction in systolic blood pressure [−3.5 mmHg (95% CI: −7.5 to 0.5)] compared to those with controlled hypertension [−1.3 mmHg (95% CI: −4.1 to 0.5)]. Exploratory analysis showed that the mean difference of the measurements of the CVRM-Box was larger than the office-based difference in measurements over the 12-month period (see Supplementary material online, *Table S3*).

#### CVRM-box measurements

At 12 months, 144 patients (59%) continued measuring their blood pressure through the final four weeks. Compared to baseline, systolic blood pressure decreased from 138.9 ± 15.4 mm Hg to 131.8 ± 11.1 mm Hg [adjusted mean difference: −5.5 mm Hg (95% CI, −7.6 to −3.3); *P* < 0.001]. Diastolic blood pressure declined from 85.7 ± 10.0 mm Hg to 81.5 ± 7.0 mm Hg [adjusted mean difference: −3.8 mm Hg (95% CI, −5.1 to −2.4); *P* < 0.001] (*Table 2*).

### Secondary outcome: CVRM-box and office-based weight measurements and physical activity

#### Office-based measurements

A greater reduction in weight was observed in the intervention group compared to the control group (reduction in weight –1.1 kg (−2.1 to −0.2), in the intervention group, - 0.3 kg [0.0 to −0.6in de control group, adjusted mean difference: –0.9 kg (95% CI, −1.6 to −0.2); *P* = 0.01]. Similarly, BMI decreased with –0.4 kg/m² (−0.7 to −0.1) in the intervention group and –0.1 kg/m² (−0.2 to 0.0) in the control group, adjusted mean difference: −0.3 kg/m² [95% CI, −0.5 to −0.01]; *P* = 0.007.

#### CVRM-box measurements

There was a reduction in weight from 90.0 ± 16.7 kg at baseline to 86.2 ± 15.9 kg at 12 months [adjusted mean difference: −1.1 kg (95% CI, −1.7 to −0.5); *P* < 0.001]. BMI also decreased from 29.5 ± 5.0 kg/m² at baseline to 28.0 ± 4.1 kg/m² at 12 months [adjusted mean difference: −0.5 kg/m² (95% CI, −0.7 to −0.2); *P* < 0.001]. Steps per day decreased slightly over the study period. Steps accounting for days tracked decreased from 4622 [3185–6269] at baseline to 4063 [3128–6237] at 12 months [adjusted mean difference: 298 steps (−597 to 20); *P* = 0.077]. When accounting for both days tracked and hours worn, steps changed from 4610 [3266–5519] (*n* = 88) at baseline to 3819 [2870–5175] at 12 months [adjusted mean difference: 48 steps (−442 to 590); *P* = 0.855] (*Table 2*).

### Medication changes

The CVRM-Box group demonstrated a greater increase in the number of antihypertensive medications compared to the control group over 12 months (*Table 3*). At baseline, participants in the intervention group used a mean of 1.44 ± 1.08 antihypertensive drugs per person, which increased to 1.56 ± 1.10 at 12 months. In contrast, the control group had a mean of 1.15 ± 0.98 antihypertensive drugs at baseline and 1.17 ± 0.98 at 12 months, resulting in an adjusted mean difference of 0.12 [95% CI: 0.06 to 0.23]; *P* = 0.04. Additionally, the defined daily dose (DDD) of angiotensin-converting enzyme inhibitors and angiotensin II receptor blockers increased more at 12 months compared to the control group [DDD adjusted mean difference: 0.09 (95% CI: 0.02 to 0.15); *P* < 0.001]. However, the DDD of thiazide diuretics and beta-blockers significantly decreased (DDD adjusted mean difference: −0.03 [95% CI: −0.07 to 0.00]; *P* = 0.04, and DDD adjusted mean difference: −0.11 [95% CI: −0.17 to −0.05]; *P* < 0.001, respectively), leading to an adjusted mean difference in total DDD of: 0.03 [95% CI: −0.01 to 0.06]; *P* = 0.17).

**Table 3:** Medication outcomes at baseline and 12 months

	Baseline mean ± SD	12 months mean ± SD	mean difference (95% CI, *P*-value)	Intervention vs. control group [mean difference (95% CI), *P* value]
**Number of antihypertensive drugs**				
**Intervention group**	1.44 ± 1.08;	1.5 ± 1.10;	0.13 (0.04 to 0.21), ***P*** **<** **0.01**	0.12 (0.06 to 0.23), ***P*** **=** **0.04**
	*n* = 220	*n* = 220		
**Control group**	1.15 ± 0.98;	1.17 ± 0.98;	0.02 (−0.03 to 0.07), *P* = 0.44	
	*n* = 965	*n* = 965		
**DDD overall**				
**Intervention group**	0.98 ± 0.98;	1.01 ± 0.44	0.03 (0.00 to 0.11), *P* = 0.37	−0.03 (−0.01 to 0.06), *P* = 0.17
	*n* = 220	*n* = 220		
**Control group**	1.01 ± 0.53;	1.01 ± 0.52;	−0.00 (−0.01 to 0.01), *P* = 0.99	
	*n* = 965	*n* = 965		
**DDD beta-blocker**				
**Intervention group**	0.26 ± 0.39;	0.21 ± 0.26;	−0.05 (−0.11 to 0.01), *P* = 0.12	−0.11 (−0.17 to −0.05), ***P*** **<** **0.001**
	*n* = 220	*n* = 220		
**Control group**	0.46 ± 0.29;	0.46 ± 0.29;	−0.00 (−0.01 to 0.01), *P* = 0.86	
	*n* = 965	*n* = 965		
**DDD of thiazide and related diuretics**				
**Intervention group**	0.65 ± 0.25	0.63 ± 0.28;	−0.02 (−0.07 to 0.03), *P* = 0.45	−0.03 (−0.07 to −0.00), ***P*** **=** **0.04**
	*n* = 220	*n* = 220		
**Control group**	0.63 ± 0.26;	0.64 ± 0.28;	−0.00 (−0.01 to 0.01), *P* = 0.85	
	*n* = 965	*n* = 965		
**DDD of angiotensin-converting-enzyme inhibitors and angiotensin II blockers**				
**Intervention group**	1.39 ± 0.80;	1.44 ± 0.79;	0.08 (−0.02 to 0.19), *P* = 0.11	0.09 (0.02 to 0.15), ***P*** **<** **0.001**
	*n* = 220	*n* = 220		
				
**Control group**	0.98 ± 0.98;	0.98 ± 0.98;	−0.01 (−0.01 to 0.03), *P* = 0.41	
	*n* = 965	*n* = 965		
**DDD of calcium antagonists**				
**Intervention group**	1.31 ± 0.49;	1.35 ± 0.51;	0.08 (0.01 to 0.15), ***P*** **<** **0.03**	0.09 (0.02 to 0.15), ***P*** **<** **0.001**
	*n* = 220	*n* = 220		
**Control group**	1.30 ± 0.52;	1.30 ± 0.52;	0.01 (−0.01 to 0.02), *P* = 0.35	
	*n* = 965	*n* = 965		

### Healthcare utilization

The CVRM-Box was associated with reduced healthcare utilization (*Table 4*). The intervention group had fewer total consultations per person compared to the control group (3.1 [SE 0.06] vs. 3.8 [SE 0.04]; rate ratio [RR] = 0.82 [95% CI, 0.78 to 0.95], *P* < 0.001) and fewer short (<20 min) GP consultations (1.3 [SE 0.04] vs. 2.0 [SE 0.08]; RR = 0.81 [95% CI, 0.67 to 0.95], *P* < 0.001). The difference in long (>20 min) GP consultations was small (1.2 [SE 0.04] vs. 1.0 [SE 0.01]; RR = 1.09 [95% CI, 0.96 to 1.22]). E-consultations (defined as digital written communications through a secure platform connected to the patient’s electronic medical record, which can be initiated by either the patient or the doctor^18^) were lower in the intervention group (0.6 [SE 0.09] vs. 0.8 [SE 0.10]; RR = 0.76 [95% CI, 0.56 to 0.96]).

**Table 4:** Consultation outcomes by study group

	Intervention group (mean, SE)	Control group (mean, SE)	Fully adjusted model rate ratio (95% CI), *P*
	(*n* = 245)	(*n* = 976)	
Number of total consultations mean (per person per year)	3.1 (0.06)	3.8 (0.04)	0.82 (0.78 to 0.95), *P* = 0.002
Number of short (<20 min) GP consultations, mean (per person)	1.3 (0.2)	2.0 (0.08)	0.81 (0.67 to 0.95), *P* = 0.01
Number of long (20 min) GP consultations, mean (per person)	1.2 (0.04)	1.0 (0.01)	1.09 (0.96 to 1.22), *P* = 0.167
Number of e-consultations*, mean (per person)	0.6 (0.09)	0.8 (0.1)	0.76 (0.56 to 0.96), *P* = 0.02

^*^ **e-consultations: e-mail, telephone and video consultations**

## Discussion

To our knowledge, this is the first study for high-risk cardiovascular patients in primary care to evaluate a multicomponent remote monitoring intervention, the CVRM-Box, comprising a blood pressure monitor, digital weight scale, activity tracker, and smartphone app. Home measurements demonstrated a reduction in systolic blood pressure, whereas office-based measurements showed no additional reduction compared to matched controls. However, the CVRM-Box group had a significantly higher proportion of patients with controlled blood pressure and lower weight and BMI than those receiving usual care at the end of follow-up. There was no change in physical activity observed. Additionally, the CVRM-Box was associated with fewer consultations for GPs and PNs.

### Comparison with literature

Our findings are consistent with, yet also differ from, two recent large RCTs evaluating remote monitoring for hypertension in primary care.^5,6^ While both studies focused exclusively on patients with uncontrolled hypertension, resulting in higher baseline blood pressure, our study included both controlled and uncontrolled patients. By incorporating shared decision-making, we aimed to provide equitable care for the entire CVRM population. This broader approach may reduce unnecessary visits for both groups, lowering healthcare workload and minimizing patients’ waiting times, travel burdens, and consultation durations. Furthermore, unlike prior studies, self-titration was not possible in our study; instead, the PN was responsible for this task. Previous studies linked reductions in blood pressure to increased use of antihypertensive drugs,^6,19^ we also observed such an increase in our study. Remote monitoring might provide earlier insights into blood pressure values, enabling timely intervention and potentially preventing clinical inertia, which is estimated to contribute to up to 80% of myocardial infarctions and strokes.^20^ Additionally, the exploratory analysis revealed greater decreases in systolic blood pressure in CVRM-Box measurements compared to office-based measurements, likely due to white coat hypertension,^21^ where home measurements decline more rapidly than office measurements. This finding supports the recommendation of home blood pressure measurements for optimizing blood pressure control as provided by the 2024 ESC Guidelines,^22^ as it minimizes variability and provides a more reliable assessment of true blood pressure levels.^23^

In recent years, remote weight monitoring has been a key element in obesity management, consistently associated with weight loss.^24^ However, most studies have focused on overweight and obese patients rather than those at high cardiovascular risk.^24^ One exception is an RCT involving 65 overweight or obese patients with a high risk of cardiovascular disease, where a 12-month intervention using a smart scale, accelerometer, and web-based support led to a weight difference of −1.89 kg between the intervention and control groups.^25^ Our study, in contrast, showed a smaller reduction of −0.9 kg, likely due to the absence of a dedicated weight loss programme and because not all participants were overweight. Incorporating targeted weight reduction programmes may improve outcomes.^26,27^

In contrast to previous studies, no significant change in physical activity was observed in our study.^28,29^ However, this finding should be interpreted with caution due to the considerable proportion of missing data, partly resulting from participants using their own activity trackers or encountering technical issues with Bluetooth connectivity. As shown in previous studies, additional behavioural components such as targeted feedback or coaching enhance engagement and increase daily step counts.^29^ This suggests that combining remote monitoring with personalized behavioural support could further stimulate physical activity. Future research should explore how these motivational elements can be sustainably integrated into digital cardiovascular risk management programmes.

Our study showed a reduction in consultation frequency, specifically short consultations (<20 min) by the PN or GP. Unlike previous studies that either overlooked healthcare utilization or reported increased consultation rates,^6,30–32^ our findings revealed a significant decrease in both total and short consultations. This reduction likely from how the care pathway was structured within the practice by the intervention, which included limiting visits to one annually and additional visits only for specific issues (e.g. elevated blood pressure or patient concerns). Most queries were managed via email or research assistant support, suggesting that remote monitoring optimizes resource allocation and improves healthcare efficiency.

Lastly, subgroup analyses revealed a sex-specific difference, with women using the CVRM-Box demonstrating a greater reduction in blood pressure compared with men, who derived only minimal benefit. This observation aligns with previously reported sex-related differences in hypertension management and treatment response within primary care in the Netherlands. This research showed that although men and women receive a comparable number of antihypertensive prescriptions, women are more frequently treated with β-blockers and diuretics, tend to receive lower dosages, and more often achieve target blood pressure levels.^33^ These variations in pharmacological management and physiological response may, at least in part, account for the more pronounced effect observed among women in the present study.

### Limitations

Several limitations must be considered when interpreting this study. First, the mean age of participants is lower than that typically found in CVRM populations, which may impact generalisability. The inclusion criteria, based on minimal technical skills required for smartphone use, likely contributed to the younger average age. Our consortium is conducting a follow-up study with low-literate patients to evaluate the intervention's effectiveness across different digital and health-related skill levels. Second, while the intervention demonstrated short-term efficacy, its long-term (10-year) impact on cardiovascular risk remains uncertain, warranting further research. Thirdly, our statistical approach—a linear mixed-effects model with a random intercept and fixed slope—may not fully capture within-patient and practice variability due to the absence of a random slope. However, this method was chosen based on the assumption of consistent effects across practices with similar implementation and educational investments. Notably, analysis showed no significant differences between the models, suggesting the fixed effects were sufficient to accurately represent the data. Finally, it is possible that the difference in observed events in the CVRM-Box measurements can be partly explained by measurement artifacts or regression to the mean.^34^ In the absence of a randomized and concurrent control group, the observed effects may reflect natural variability over time rather than true intervention effects.

Despite these limitations, the study offers valuable insights into CVRM, underscoring the importance of cautious interpretation and further research to address these methodological issues. Its strong external validity enhances generalisability to real-world primary care. Additionally, integrating the intervention into a robust digital infrastructure enabled efficient remote data exchange and direct EHR access for healthcare providers.

### Implications for research and practice

As remote monitoring becomes more integrated into clinical practice and healthcare professionals recognize its benefits,^35^ along with previous studies showing similar results,^5,6,32^ our current study provides evidence on clinical outcomes and healthcare utilization to support and expand its large-scale implementation in daily practice. Our digital health infrastructure fosters the creation of a Learning Health System (LHS), which leverages routine data for real-time feedback to enhance care processes and personalize patient care, as we aimed to achieve in our study.^36,37^ Moreover, by empowering patients to manage their health with remote monitoring tools, we can alleviate some of the burden on healthcare professionals. Finally, it is crucial to assess whether the right patient populations are targeted to optimize implementation strategies and cost-effectiveness. Future research should focus on refining remote monitoring interventions to better meet the specific needs of different patient groups. Currently, our research addresses these issues by allocating different patient groups to tailored devices within the CVRM-Box and testing its cost-effectiveness.

## Conclusions

The CVRM-Box efficiently enhances CVRM in primary care. While office-based blood pressure measurements showed no additional decrease compared to the control group, the CVRM-Box measurements revealed a reduction in blood pressure. Additionally, the proportion of patients achieving controlled blood pressure increased, and weight reduction was observed in both office-based compared to the control group and CVRM-Box measurements. The intervention also resulted in more prescribed antihypertensive medications and fewer consultations.

## Lead author biography


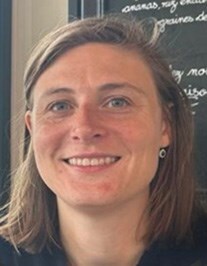
*van Hattem, MD, is a PhD candidate studying the effect and implementation of remote patient management in primary care. She combines her PhD research with her training as a general practitioner, focusing on improving the integration of digital health innovations into everyday clinical practice.


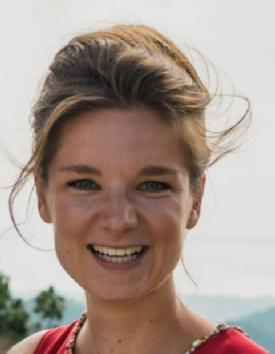
*Rakers, MD, is pursuing a PhD focused on leveraging data-driven digital health interventions to shift healthcare from reactive to proactive models and is currently training as a sports medicine resident at Utrecht University Medical Center. In parallel with her PhD, she founded **HealthInnovaitors**, an initiative dedicated to advancing the adoption of artificial intelligence to improve healthcare delivery and patient outcomes.

*both contributed equally

## Supplementary Material

ztag014_Supplementary_Data

## Data Availability

The data underlying this article will be shared on reasonable request to the corresponding author.
