# Supplementary material for: Effectiveness of remote monitoring for patients with a high risk of cardiovascular disease: a 12-month matched cohort study in primary care
Source: Eur Heart J Digit Health. 2026 Jan 22;7(2):ztag014. doi: 10.1093/ehjdh/ztag014 (PMC12907927; doi:10.1093/ehjdh/ztag014)
Supplement: ztag014_Supplementary_Data [file ztag014_supplementary_data.docx]

**Supplementary Online Content**

**Figure 1 - Intervention processes…………………………………………………………………………………………………………………………………………………………………………………….2**

**Figure 2 - Flowchart of The Box matched cohort study…………………………………………………………………………………………………………………………………………………..3**

**Table 1 – STROBE checklist……………………………………………………………………………………………………………………………………………………………………………………………..4**

**Table 2 – Sensitivity analysis for the systolic and diastolic blood pressure ……………………………………………………………………………………………..…………….………..5**

**Table 3 – Comparison reduction CVRM-Box measurements vs office-based measurements over 12 months……………………………………………………………..……5**

**Figure 1 – Intervention processes**


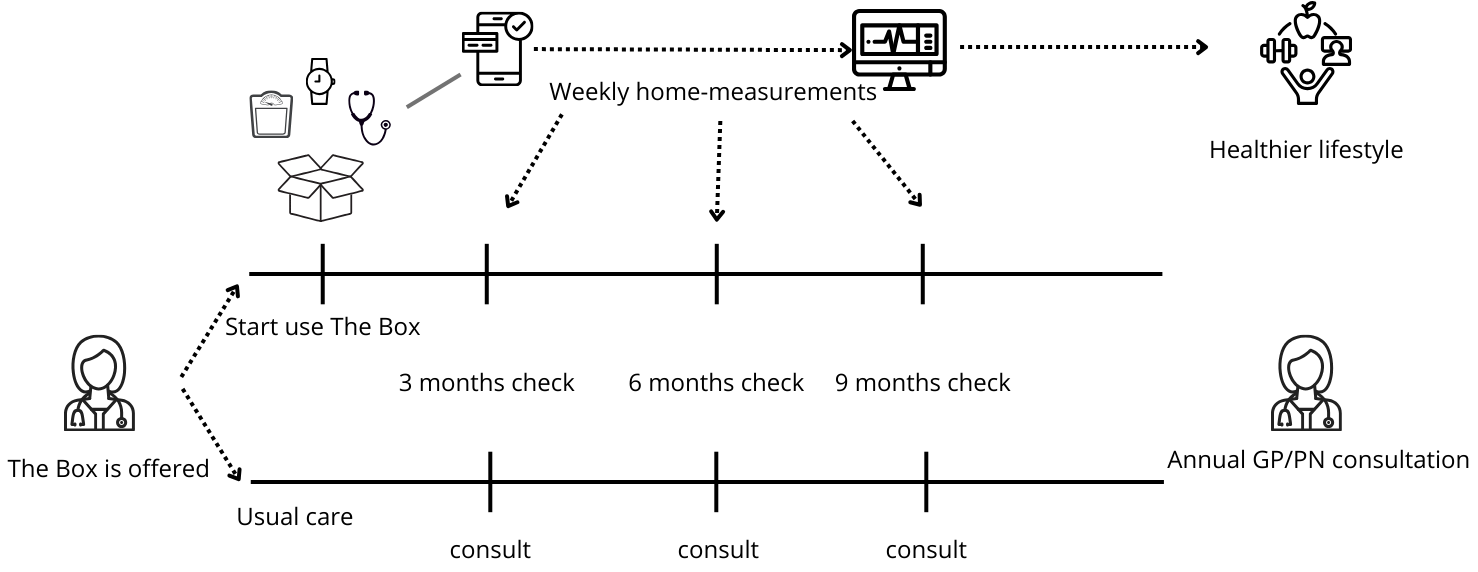


**Figure 2 - Flowchart of The Box matched cohort study**

*
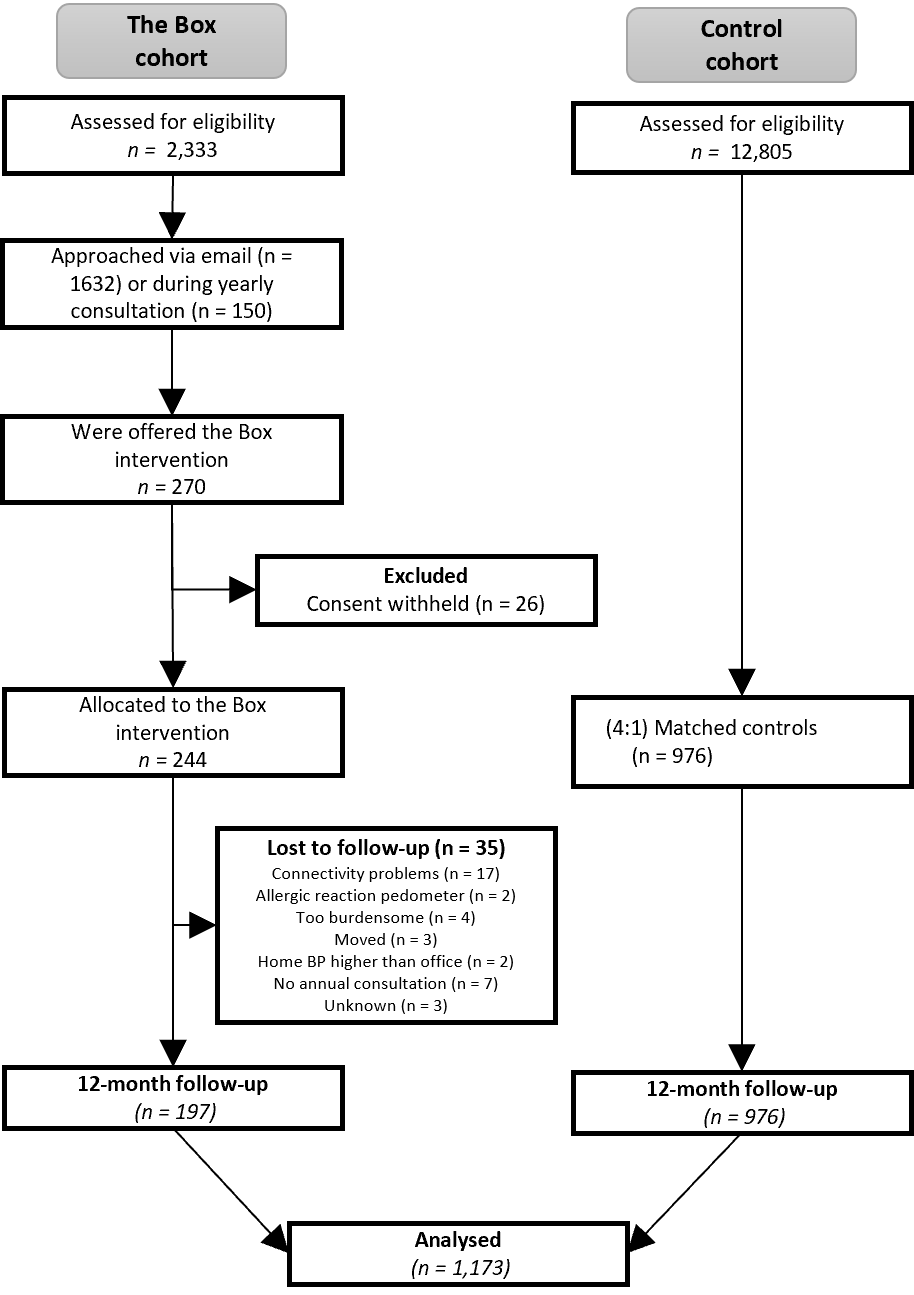
*

**Table 1 – STROBE checklist**

STROBE Statement—checklist of items that should be included in reports of observational studies

|  | Item No. | Recommendation | Page  No. | Relevant text from manuscript |
| --- | --- | --- | --- | --- |
| **Title and abstract** | 1 | (*a*) Indicate the study’s design with a commonly used term in the title or the abstract | 1 | A 12-month matched cohort study in primary care |
|  |  | (*b*) Provide in the abstract an informative and balanced summary of what was done and what was found | 2 | In this matched cohort study, patients with a >5% 10-year CVD mortality risk in primary care (2020–2024) were compared to propensity score-matched controls over 12 months. The CVRM-Box included smartphone-connected devices (blood pressure monitor, weighing scale, activity tracker) linked to general practitioner electronic health records.  Compared to controls, the intervention group showed modest reductions in office-measured systolic (-1.1 mmHg [95% CI, -3.7 to -1.5]; p = 0.39) and diastolic blood pressure (-0.04 mmHg [95% CI, -1.6 to 1.5]; p = 0.96). Sensitivity analyses yielded similar results. However, CVRM-Box assessments showed reductions in systolic (-5.5 mmHg [95% CI, -7.6 to -3.3]; p < 0.001) and diastolic blood pressure (-3.8 mmHg [95% CI, -5.1 to -2.4]; p < 0.001). The intervention group also experienced greater reductions in weight (-0.9 kg [95% CI, -1.6 to -0.2]; p = 0.01) and BMI (-0.3 kg/m² [95% CI, -0.5 to -0.01]; p =0.007). Additionally, antihypertensive medication use increased (0.12 [95% CI, 0.06 to 0.23]; p = 0.04), while consultation frequency decreased (rate ratio 0.82; p = 0.002). |
| Introduction | | | |  |
| Background/rationale | 2 | Explain the scientific background and rationale for the investigation being reported | 4 | Although cardiovascular disease mortality has declined over recent decades, rising obesity rates, an aging population, and healthcare staff shortages are increasing morbidity and healthcare burden 1,2. Therefore, exploring efficient and sustainable methods for delivering (preventive) care, such as remote monitoring of blood pressure, weight, and physical activity, is essential. These approaches help mitigate cardiovascular risk factors, including hypertension and overweight, while reducing strain on the healthcare system 3-6.  Previous studies have shown that regular remote monitoring provides a more accurate reflection of blood pressure than sporadic office-based measurements 7,8. Additionally, randomised controlled trials (RCTs) have shown that remote monitoring has been shown to significantly reduce blood pressure in patients with poorly controlled (> 140/90mmHg) hypertension compared to standard care 5,6. However, most eHealth interventions for high-risk primary care patients have focused primarily on hypertension management, overlooking other crucial aspects of cardiovascular risk, such as body weight. Moreover, their impact on healthcare provider workload is often not assessed. |
| Objectives | 3 | State specific objectives, including any prespecified hypotheses | 4 | This study aims to examine the effect of remote monitoring using the CVRM-Box in a primary care setting to improve blood pressure control, weight management, medication prescriptions and primary care consultation frequency. |
| Methods | | | |  |
| Study design | 4 | Present key elements of study design early in the paper | 5 | This matched cohort study was |
| Setting | 5 | Describe the setting, locations, and relevant dates, including periods of recruitment, exposure, follow-up, and data collection | 5 - 6 | conducted from June 2020 to June 2024 among six primary care practices in healthcare centers in the Leiden region, The Netherlands. atients were recruited from six practices either during their annual CVRM consultation, where healthcare providers introduced the CVRM-Box, or through an information session at a local community center. In both cases, patients could complete informed consent online and access detailed study information.  A control group was selected using propensity score matching in the Extramural LUMC Academic Network (ELAN) database, a regional integrative population-based data infrastructure 15.. |
| Participants | 6 | (*a*) *Cohort study*—Give the eligibility criteria, and the sources and methods of selection of participants. Describe methods of follow-up |  | Patients enrolled in care programs for CVRM or diabetes mellitus (DM), indicating a high risk of cardiovascular disease (defined as >5% 10-year mortality according to the SCORE Table) 14, or those with established cardiovascular disease (acute coronary syndrome, percutaneous coronary intervention, stroke, and peripheral arterial disease) were eligible to participate in this study. Additional inclusion criteria included being 18 years or older, owning a smartphone, having internet access at home, and being proficient in Dutch. Exclusion criteria included pregnancy and unwillingness to sign informed consent. A maximum of 270 participants could, with selection based on the availability of CVRM-Boxes within participating general practices and patients’ willingness to participate. |
|  |  | (*b*) *Cohort study*—For matched studies, give matching criteria and number of exposed and unexposed | 6 | A 1:4 propensity score matching was performed using logistic regression to calculate the propensity score and with nearest neighbour matching (R-studio, MatchIt) based on several baseline variables, including demographics (age, sex, social demographic status), comorbidities (diabetes mellitus, stroke, myocardial infarction), clinical values (systolic and diastolic blood pressure, BMI, number of antihypertensives), and laboratory results (eGFR, HDL, LDL, total cholesterol, HbA1c). |
| Variables | 7 | Clearly define all outcomes, exposures, predictors, potential confounders, and effect modifiers. Give diagnostic criteria, if applicable | 7 | The primary outcomes were CVRM-Box and office-based blood pressure measurements over a 12-month period. Office-based blood pressure was recorded by a PN using a calibrated blood pressure monitor during the standard annual consultation. This included a baseline measurement, a follow-up after 12 months, and any additional measurements from interim consultations.  Secondary outcomes included the percentage of patients with controlled blood pressure (<140 mmHg) at the GP office after 12 months, changes in body weight measured by the CVRM-Box and at the GP practice, changes in physical activity measured by the CVRM-Box, changes in antihypertensive prescriptions (number and defined daily dose [DDD]), healthcare utilisation measured by consultation frequency (defined as consultations within CVRM care) and the comparison of systolic blood pressure reduction between CVRM-Box and office-based measurements. |
| Data sources/ measurement | 8* | For each variable of interest, give sources of data and details of methods of assessment (measurement). Describe comparability of assessment methods if there is more than one group |  | . |
| Bias | 9 | Describe any efforts to address potential sources of bias |  | Covariates included sex, baseline blood pressure, baseline BMI, age and cardiovascular disease history. |
| Study size | 10 | Explain how the study size was arrived at |  |  |

Continued on next page

| Quantitative variables | 11 | Explain how quantitative variables were handled in the analyses. If applicable, describe which groupings were chosen and why | | 8 | Please see description below | |
| --- | --- | --- | --- | --- | --- | --- |
| Statistical methods | 12 | (*a*) Describe all statistical methods, including those used to control for confounding | | 8 -9 | For CVRM-Box measurements, the baseline was defined as the average of the first two weeks of measurements, and 12-month values as the average of the last four weeks. A linear mixed-effects (LME) (R-studio version 4.2, lme4) model accounted for repeated measures, with random intercepts for GP practices and patients, and fixed effects for time and intervention. Covariates included sex, baseline blood pressure, baseline BMI, age and cardiovascular disease history. An interaction term between time and intervention assessed treatment effects, and missing CVRM-Box data were imputed using last observation carried forward (LOCF), assuming stability of blood pressure over time and preventing overestimation of the Box’s effect. For the activity tracker data, only patients with at least four days of available measurements per week were included 16.  For office-based measurements, the same LME model was used to compare the intervention and control group. To assess the robustness of our findings, we conducted sensitivity analyses using different approaches to handle missing data, including complete case analysis, last observation carried forward (LOCF), and multiple imputation by chained equations (MICE). Additionally, interaction terms (time * intervention and subgroup) were used to evaluate the intervention's effect across subgroups: age (<70 vs. >70 years), sex, baseline office systolic blood pressure (<140/90 vs. >140/90 mmHg), and BMI (normal weight: BMI <25, overweight: BMI 25-30, and obesity: BMI >30).  For secondary outcomes, differences in weight and BMI between baseline and one year were assessed using a similar LME model, with adjustments for the same covariates, for both CVRM-Box and office measurements. Changes in the number of antihypertensive prescriptions and DDD from baseline to 12 months were analysed using linear regression. Consultation frequency was assessed with negative binomial regression and group differences were reported as adjusted rate ratios (RRs) with 95% CI. An exploratory analysis compared CVRM-Box and office-based measurements using a paired t-test in patients with both measurements at T0 and T12. | |
|  |  | (*b*) Describe any methods used to examine subgroups and interactions | | 8 | Additionally, interaction terms (time * intervention and subgroup) were used to evaluate the intervention's effect across subgroups: age (<70 vs. >70 years), sex, baseline office systolic blood pressure (<140/90 vs. >140/90 mmHg), and BMI (normal weight: BMI <25, overweight: BMI 25-30, and obesity: BMI >30). | |
|  |  | (*c*) Explain how missing data were addressed | | 8 | and missing CVRM-Box data were imputed using last observation carried forward (LOCF), assuming stability of blood pressure over time and preventing overestimation of the Box’s effect | |
|  |  | (*d*) *Cohort study*—If applicable, explain how loss to follow-up was addressed | |  |  | |
|  |  | (*e*) Describe any sensitivity analyses | | 8 | To assess the robustness of our findings, we conducted sensitivity analyses using different approaches to handle missing data, including complete case analysis, last observation carried forward (LOCF), and multiple imputation by chained equations (MICE). | |
| Results | | | | | | |
| Participants | 13* | (a) Report numbers of individuals at each stage of study—eg numbers potentially eligible, examined for eligibility, confirmed eligible, included in the study, completing follow-up, and analysed | | 10 | Of the 2333 individuals assessed across six practices, 1832 (79%) were approached via email (89%) and during their yearly consultation (11%). A maximum of 270 patients could participate in the study. Among these, 26 (1%) withheld their consent (Supplementary Figure 2) resulting in 244 allocated to the intervention. The primary reasons for exclusion included connectivity or technical problems, 17 patients), the study being too burdensome (four patients), and no annual consultation at 12 months (seven patients). A total of 976 matched control patients were included for comparison | |
|  |  | (b) Give reasons for non-participation at each stage | | 10 | See tekst above | |
|  |  | (c) Consider use of a flow diagram | | 10 | Supplementary Figure 2 | |
| Descriptive data | 14* | (a) Give characteristics of study participants (eg demographic, clinical, social) and information on exposures and potential confounders | | 10 | Baseline characteristics after the propensity score matching are summarised in Table 1 and the corresponding balance plot is visualised in Figure 2. The mean (SD) age was 62.4 (11) years in the CVRM-Box group and 63.2 (10.4) years in the control group, with approximately 55% male participants. Mean baseline blood pressure was similar between groups (140/85 mmHg). | |
|  |  | (b) Indicate number of participants with missing data for each variable of interest | | 10 | After 12 months, the primary outcome data were available from 197 (81%) participants in the CVRM-Box group and 894 (92 %) in the control group | |
|  |  | (c) *Cohort study*—Summarise follow-up time (eg, average and total amount) | |  |  | |
| Outcome data | 15* | *Cohort study*—Report numbers of outcome events or summary measures over time | | 10 | After 12 months, the primary outcome data were available from 197 (81%) participants in the CVRM-Box group and 894 (92 %) in the control group | |
|  |  |  |  | | |  |
|  |  |  |  | | |  |
| Main results | 16 | (*a*) Give unadjusted estimates and, if applicable, confounder-adjusted estimates and their precision (eg, 95% confidence interval). Make clear which confounders were adjusted for and why they were included | | 10-13 | See table 2-4, page 23-26 | |
|  |  | (*b*) Report category boundaries when continuous variables were categorized | | Not applicable |  | |
|  |  | (*c*) If relevant, consider translating estimates of relative risk into absolute risk for a meaningful time period | | Not applicable |  | |

Continued on next page

| Other analyses | 17 | Report other analyses done—eg analyses of subgroups and interactions, and sensitivity analyses | 10-13 |  |
| --- | --- | --- | --- | --- |
| Discussion | | | | |
| Key results | 18 | Summarise key results with reference to study objectives | 14 | To our knowledge, this is the first study for high-risk cardiovascular patients in primary care to evaluate a multicomponent remote monitoring intervention, the CVRM-Box, comprising a blood pressure monitor, digital weight scale, activity tracker, and smartphone app. Home measurements demonstrated a reduction in systolic blood pressure, whereas office-based measurements showed no additional reduction compared to matched controls. However, the CVRM-Box group had a significantly higher proportion of patients with controlled blood pressure and lower weight and BMI than those receiving usual care at the end of follow-up. There was no change in physical activity observed. Additionally, the CVRM-Box was associated with fewer consultations for GPs and PNs. |
| Limitations | 19 | Discuss limitations of the study, taking into account sources of potential bias or imprecision. Discuss both direction and magnitude of any potential bias | 16-17 | Several limitations must be considered when interpreting this study. Firstly, the mean age of participants is lower than that typically found in CVRM populations, which may impact generalisability. The inclusion criteria, based on minimal technical skills required for smartphone use, likely contributed to the younger average age. Our consortium is conducting a follow-up study with low-literate patients to evaluate the intervention's effectiveness across different digital and health-related skill levels. Secondly, while the intervention demonstrated short-term efficacy, its long-term (10-year) impact on cardiovascular risk remains uncertain, warranting further research. Thirdly, our statistical approach—a linear mixed-effects model with a random intercept and fixed slope—may not fully capture within-patient and practice variability due to the absence of a random slope. However, this method was chosen based on the assumption of consistent effects across practices with similar implementation and educational investments. Notably, analysis showed no significant differences between the models, suggesting the fixed effects were sufficient to accurately represent the data. Finally, it is possible that the difference in observed events in the CVRM-Box measurements can be partly explained by measurement artifacts or regression to the mean Click or tap here to enter text.33. In the absence of a randomised and concurrent control group, the observed effects may reflect natural variability over time rather than true intervention effects. |
| Interpretation | 20 | Give a cautious overall interpretation of results considering objectives, limitations, multiplicity of analyses, results from similar studies, and other relevant evidence | 17 | As remote monitoring becomes more integrated into clinical practice and healthcare professionals recognise its benefits 34, along with previous studies showing similar results 5,6,31, our current study provides evidence on clinical outcomes and healthcare utilisation to support and expand its large-scale implementation in daily practice. Our digital health infrastructure fosters the creation of a Learning Health System (LHS), which leverages routine data for real-time feedback to enhance care processes and personalize patient care, as we aimed to achieve in our study 35,36. Moreover, by empowering patients to manage their health with remote monitoring tools, we can alleviate some of the burden on healthcare professionals. Finally, it is crucial to assess whether the right patient populations are targeted to optimize implementation strategies and cost-effectiveness. Future research should focus on refining remote monitoring interventions to better meet the specific needs of different patient groups. Currently, our research addresses these issues by allocating different patient groups to tailored devices within the CVRM-Box and testing its cost-effectiveness. |
| Generalisability | 21 | Discuss the generalisability (external validity) of the study results | 17 | Despite these limitations, the study offers valuable insights into CVRM, underscoring the importance of cautious interpretation and further research to address these methodological issues. Its strong external validity enhances generalisability to real-world primary care. Additionally, integrating the intervention into a robust digital infrastructure enabled efficient remote data exchange and direct EHR access for healthcare providers. |
| Other information | |  | | |
| Funding | 22 | Give the source of funding and the role of the funders for the present study and, if applicable, for the original study on which the present article is based | 19 | This study was funded by a grant from Innovative Medical Devices Initiative (IMDI), LSHM21009 and Zorginstituut Nederland ‘Subsidieregeling leren gebruiken van uitkomstinformatie voor Samen beslissen 2021-2025’, 3463471-1039183-PZO |

*Give information separately for cases and controls in case-control studies and, if applicable, for exposed and unexposed groups in cohort and cross-sectional studies.

**Note:** An Explanation and Elaboration article discusses each checklist item and gives methodological background and published examples of transparent reporting. The STROBE checklist is best used in conjunction with this article (freely available on the Web sites of PLoS Medicine at http://www.plosmedicine.org/, Annals of Internal Medicine at http://www.annals.org/, and Epidemiology at http://www.epidem.com/). Information on the STROBE Initiative is available at www.strobe-statement.org.

**Table 2 – Sensitivity analysis for the systolic and diastolic blood pressure**

**Table 3 - Comparison reduction CVRM-Box measurements vs office-based measurements over 12 months**

|  | **Baseline** | **12 monts** | **Difference** | **Mean difference** |
| --- | --- | --- | --- | --- |
| **Systolic** **bloodpressure** | | | |  |
| **CVRM-Box measurements**  **N= 110** | 142.3 ± 16.7 | 133.6 ± 12.9 | -8.7 ± 16.8 | -5.6 [1.4-9.7], p 0.001 |
| **Office measurements**  **N=110** | 140.3 ± 16.3 | 137.2 ± 15.7 | -3.05 ± 15.4 |  |
| **Diastolic bloodpressure** | | | |  |
| **CVRM-Box measurements**  **N=110** | 86.5 ± 8.9 | 81.3 ± 7.1 | -5.1 ± 8.0 | -3.5 [ 1.0 – 6.1] , p 0.001 |
| **Office measurements**  **N=110** | 83.4 ± 10.7 | 81.8 ± 10.4 | -1.7 ± 10.7 |  |
